# Effects of neuromuscular training compared to classic strength-resistance training in patients with acute coronary syndrome: A study protocol for a randomized controlled trial

**DOI:** 10.1371/journal.pone.0243917

**Published:** 2020-12-23

**Authors:** Francisco José Ferrer-Sargues, Óscar Fabregat-Andrés, Isabel Martínez-Hurtado, Pablo Salvador-Coloma, Francisco José Martínez-Olmos, Marta Lluesma-Vidal, Gemma Biviá-Roig, María José Segrera-Rovira, María Dolores Arguisuelas, Noemí Valtueña-Gimeno

**Affiliations:** 1 Department of Physiotherapy, Universidad Cardenal Herrera CEU, CEU Universities, Valencia, Spain; 2 Department of Medicine, Universidad Cardenal Herrera CEU, CEU Universities, Valencia, Spain; 3 Department of Cardiology, Hospital IMED, Valencia, Spain; 4 Department of Nursing, Universidad Cardenal Herrera CEU, CEU Universities, Valencia, Spain; Kurume University School of Medicine, JAPAN

## Abstract

The aim of the present clinical trial is to evaluate the effectiveness of neuromuscular versus classical strength-resistance training as part of a cardiac rehabilitation programme in patients following acute coronary syndrome. The study is designed as a double-blinded, randomised, and controlled clinical trial. Thirty participants suffering from acute coronary syndrome who meet our inclusion criteria will be recruited by a private tertiary hospital. The intervention group will follow 20 sessions of a cardiac rehabilitation programme divided into two parts: aerobic training and neuromuscular strength-resistance training. The control group will complete the same aerobic training as well as a classical strength-resistance training workout programme. The primary outcome of the study will be the mean difference in change from baseline in the Incremental Shuttle Walking Test. The secondary outcomes will be the cardiorespiratory fitness of the patients (assessed by means of the Chester Step Test), lower-limb performance (assessed with the 30-Second Chair Stand Test and Single-Leg Squat Test), lower-limb strength (hip flexor handheld dynamometry), sexual dysfunction assessment (Sex Health Inventory for Men) and quality of life (EQ-5D-5L). This work will provide evidence for the effectiveness of a neuromuscular versus a classic strength-training programme in terms of cardiorespiratory fitness, lower-limb performance capacities and quality of life, in cardiac patients. The data obtained could lead to more effective and functional workouts which, in turn, may enhance the speed at which these patients can return to their everyday activities of life and improve the efficiency of their movement patterns and heart responses. Furthermore, patients may find neuromuscular workout routines more motivating and engaging, thus encouraging them to adopt healthier lifestyle patterns.

## Introduction

Acute coronary syndrome (ACS) is the sudden imbalance between myocardial oxygen consumption and demand (usually as the result of coronary artery obstruction), which causes an acute myocardial infarction [1]. Heart ischemic pathologies caused 29% of deaths in industrialised countries over the last decade [2] and will be the leading cause of death in 2020 [3]. This data highlights the importance of addressing this syndrome, not only from the point of view of practitioners and surgeons, but also in terms of the availability and success of cardiac rehabilitation programmes (CRPs)—multifactorial exercise-based interventions [4].

The initial objective for cardiology patients was to reduce their morbidity and mortality. However, latest advances in medicine and rehabilitation have changed the treatment framework of these patients, as now clinical focus is set in improving patient’s quality of life [5, 6]. Health-related quality of life (HRQoL) is a complex concept that is particularly useful for assessing the impact of illness on patient health status [7]. HRQoL encompasses several factors including not only physical symptoms, but also cognitive function, psychological wellbeing, and even sexual activity, as essential aspects of a healthy lifestyle [8, 9].

Quality of life is causally related to lifestyle in cardiac patients. Indeed, the major role that an physically active lifestyle plays in cardiovascular performance has been outlined in several different population types [10, 11]. Exercise-based programmes are a mainstay of health, and have a high cost-effectiveness ratio in terms of reducing the consumption of care services, and significantly improve mobility, ability to perform the daily activities of life, and levels of discomfort in patients with cardiovascular diseases [12].

CRPs have been recommended by the World Health Organization since the early 1960s [13]. They are useful for controlling cardiovascular risk factors and impact the overall factors related to HRQoL, thus reducing patient morbidity and mortality, and promoting their return to work (job reinsertion). These programmes have been proven effective, although this partially depends on their design [14, 15]. CRPs have two parts: an aerobic exercise intervention and a strength-resistance exercise programme. Although the benefits of most modalities of aerobic exercise have been studied [16], the importance of strength-resistance training and its proven effectiveness and safety for cardiac patients is also becoming increasingly obvious [17].

However, despite the recommendations of increasing strength workout in different cardiac populations, both adult and pediatric [18, 19], nowadays this phase is still subject to the aerobic one, and the vast majority of the exercises only include analytical muscle groups workout, without different levels of progressions throughout the program [20]. New types of training are now also being taken into consideration instead of the classic strength-resistance exercises, in order to increase the muscle groups involved and make a progression in complexity and intensity of them. For example, there has recently been notable interest in the study of movement dysfunction and its influence on the efficiency of everyday movements and the quality of sporting gestures. Indeed, the studies by Sahrmann *et al*. [21, 22] and Comerford *et al*. [23] outlined the key importance of the neuromuscular control of movement and the quality of movement in preventing injuries.

Training neuromuscular skills for proper movement control is not only relevant to the practice of sports, but also to other patients with pathologies such as hip or knee osteoarthritis. Thus, it is also worth exploring the benefits of neuromuscular training (NMT) for diseases other than musculoskeletal pathologies because they can help improve the general status of patients suffering from chronic diseases [24]. However, no CRPs currently incorporate NMT to improve movement control into the cardiac rehabilitation protocols prescribed to patients with ACS. Up to our knowledge, the only exception is a previous pilot study developed by our research team with a sample of ten patients with ACS undergoing a 20 week training period. Five patients conducted a NMT programme while the other five patients carried out a classic strength-resistance programme. Promising results were found in favor of the NMT group both in functional capacities and quality of life parameters (S1 Table).

The aim of this randomized controlled trial is to evaluate the short and long-term effects of neuromuscular versus classical strength-resistance training as part of a CRP in patients following ACS. Both groups of patients will perform an aerobic training programme followed either by a NMT intervention or by a classic strength-resistance training protocol (CSRT) targeting large muscle groups for the standard CRP or NMT groups, respectively. We hypothesize that the NMT will improve the cardiorespiratory fitness, functional capacities, sexual dysfunction and quality of life of patients with ACS more than the CSRT intervention.

## Materials and methods

### Study design

This study will be a double-blinded, randomised, and controlled clinical trial implemented in a university health clinic. Our study was written in accordance with Standard Protocol Items: Recommendations for Interventional Trials (SPIRIT) [25], which was aim to improve the quality of clinical trial (S1 Checklist).

### Ethical approval and registration

The design of this study conforms to the principles outlined in the Declaration of Helsinki and it was approved by the Research Ethics Committee at University CEU Cardenal Herrera (reference number CEI18/111). Participation in the study will be voluntary and will require the written informed consent from each participant. Eligible patients will be informed about all the relevant aspects of this study before starting their rehabilitation programme. The protocol was registered on the U.S. National Library of Medicine (ClinicalTrials.gov) with identifier NCT04246008 on 29 January 2020. Personal information will be collected by clinical research coordinators and stored password-protected computer to collect confidentiality.

### Participants: Recruitment and eligibility criteria

Thirty patients will participate in this study and their recruitment is expected to continue from February until July 2021. The inclusion criteria will be the following: patients aged 18–80 years, with a diagnosis of ACS with or without ST-segment elevation, a moderate or low-risk stratification according to cardiopulmonary exercise test (CPET) results and the guidelines published by the American Heart Association [26], and a medical prescription for cardiac rehabilitation.

The exclusion criteria will be the presence of any pathologies or acute conditions outlined in the American College of Sports Medicine guidelines for exercise testing and prescription with an absolute contraindication for physical exercise [27]. Other conditions leading to patient exclusion will be CPET abnormalities, severe exercise-induced arrhythmia, ST-segment depression caused by exertion, undue hypertensive responses, hypotension caused by exertion, or thoracic pain.

### Randomisation and blinding

Patients will be recruited at the Cardiology Service at a private tertiary hospital a statistician outside this research team will generate the random sequence using random number allocation software [28]. The random sequence will be concealed from all other study investigators through the entire study period. A block size of six subjects will be applied to determine the assignment into NMT or CSRT groups. To obtain a balanced within-group distribution according to sex, this variable will be blocked during the assignment. Then, the patients will be referred to the university health clinic where the physical therapist responsible for implementing this study will obtain written informed consents from all subjects and will schedule their programme sessions. Each of the patient evaluation tests will be identical and the physical therapist undertaking these tests will be blinded to the patient allocation. In addition, the patients will also be blinded to the rehabilitation type they are assigned; they will only be aware that their sessions will comprise both cardiorespiratory fitness and strength training exercises. It will be impossible to blind the physical therapists delivering the training programmes to the patient allocations because of their active role in administering these treatment sessions.

### Intervention

Intervention will comprise 20 sessions and was designed following the American College of Sports Medicine guidelines for prescribed exercise following the frequency, intensity, type, and time principles for cardiac patients [27]. Frequency will be set to twice a week. Intensity will be adjusted from CPET parameters, regulating endurance training to achieve a heart rate (HR) near VT1 at the beginning of the program, and progressively moving towards VT2 or a maximal heart rate (MHR) of 85% of peak HR. Type of intervention will include endurance and strength-resistance training. Each training session will last for 60 min, and will comprise a 10-minute warm-up, 20 minutes of cardiorespiratory fitness training, 20 minutes of strength-resistance training, finishing with 10 minutes of cool-down and stretching exercises. A total of twenty supervised sessions will be performed in harmony with the recommendations of previous studies.

Endurance phase of the CRP will be performed on a treadmill (Ergosprint^©^, Ergoline GmbH) or on a bicycle ergometer (Ergoselect200^©^, Ergoline GmbH). This work will be either continuous or intervallic, depending on the individual risk stratification of each patient. Both groups will perform both exercise types to avoid bias in their trained cardiorespiratory capacities.

Strength-resistance training will depend on the group to which the participants are assigned. The intervention in both groups has been designed to train the same muscle groups to avoid bias in upper and lower-limb performance between these groups. The CSRT group will perform general upper and lower-limb exercises which target all the large muscle groups and will progress from open-chain to closed-chain body-weighted exercises. The NMT group will complete a battery of exercises designed to improve trunk stabilisation, upper-limb dissociation from the trunk, movement patterns, muscle recruitment, and control during the range of hip and knee motions.

All subjects will be monitored during the session. Peripheral oxygen saturation (Pulsioximeter OXYM4000, Quirumed S.L.U., Spain) and HR (Polar Team H10^©^ Polar Electro OY) will be acquired continuously. Blood pressure (BP) of the patients will be recorded at the beginning of the session, after the cardiorespiratory exercises, and after the strength-resistance training section of the intervention with an Omron M6 Comfort Blood Pressure Monitor (Omron Healthcare Europe B.V, Hoofddorp, The Netherlands). Patients’ perceived exertion will be registered using a Borg CR-10 scale at the beginning, after each training phase, and at the end of each session. Training will always be led by two experienced physiotherapists. If BP exceeds the established systolic or diastolic limits, the patient will have to rest or walk at a slow pace for a few minutes; their BP will then be taken again, and if their response is found to be abnormal or unstable, the patient will be referred back to the cardiology service for re-evaluation. For the purposes of safety, the patient HR will not be allowed to exceed 85% of their MHR threshold during these training sessions.

Progression criteria for the difficulty and load of the exercises will be determined according to the HR and perceived exertion of each subject. The patient will be able to progress when they can fully complete the most demanding exercise while maintaining their established HR below the 85% MHR threshold without registering a score exceeding 4 out of 10 for dyspnea on the Borg CR-10 scale. Monitoring for protocol adherence will be performed weekly to ensure early identification of poor performance.

### Outcomes and measurement

The attending physical therapist will collect sociodemographic data from the participants in this study regarding their sex, age, marital status, education, and occupation. In addition, every patient will perform a CPET considered as the gold standard VO_2_ max value.

We will evaluate the effect of neuromuscular training compared to more traditional strength resistance training in terms of cardiorespiratory fitness, lower-limb functional performance, sexual dysfunction and quality of life. The tests and questionnaires used for these assessments are listed along with their outcome variables in Table 1.

**Table 1:** Outcomes, measures, and assessment methods that will be used in this clinical trial.

OUTCOMES	MEASURES	ASSESSMENT		
		t 1	t 2	t 3
VO^2^ max direct method	Cardipulmonary Exercise Testing	X	X	X
VO^2^ max indirect method	Incremetnal Shuttle Walking Test	X	X	X
	Chester Step Test	X	X	X
Lower-limb performance based-test	30-Second Chair Stand Test	X	X	X
	Single Leg Squat Test	X	X	X
Lower-limb strength	Hip flexor handled dynamometry	X	X	X
Sexual dysfunction	Sexual Health Inventory for Men	X	X	X
HRQoL	EQ-5D-5L	X	X	X

HRQoL Health related quality of life; EQ-5D-5L EuroQol five dimensions five levels questionnaire; t1 before intervention; t2 after intervention; t3 twelve months follow-up.

**Fig 1:**
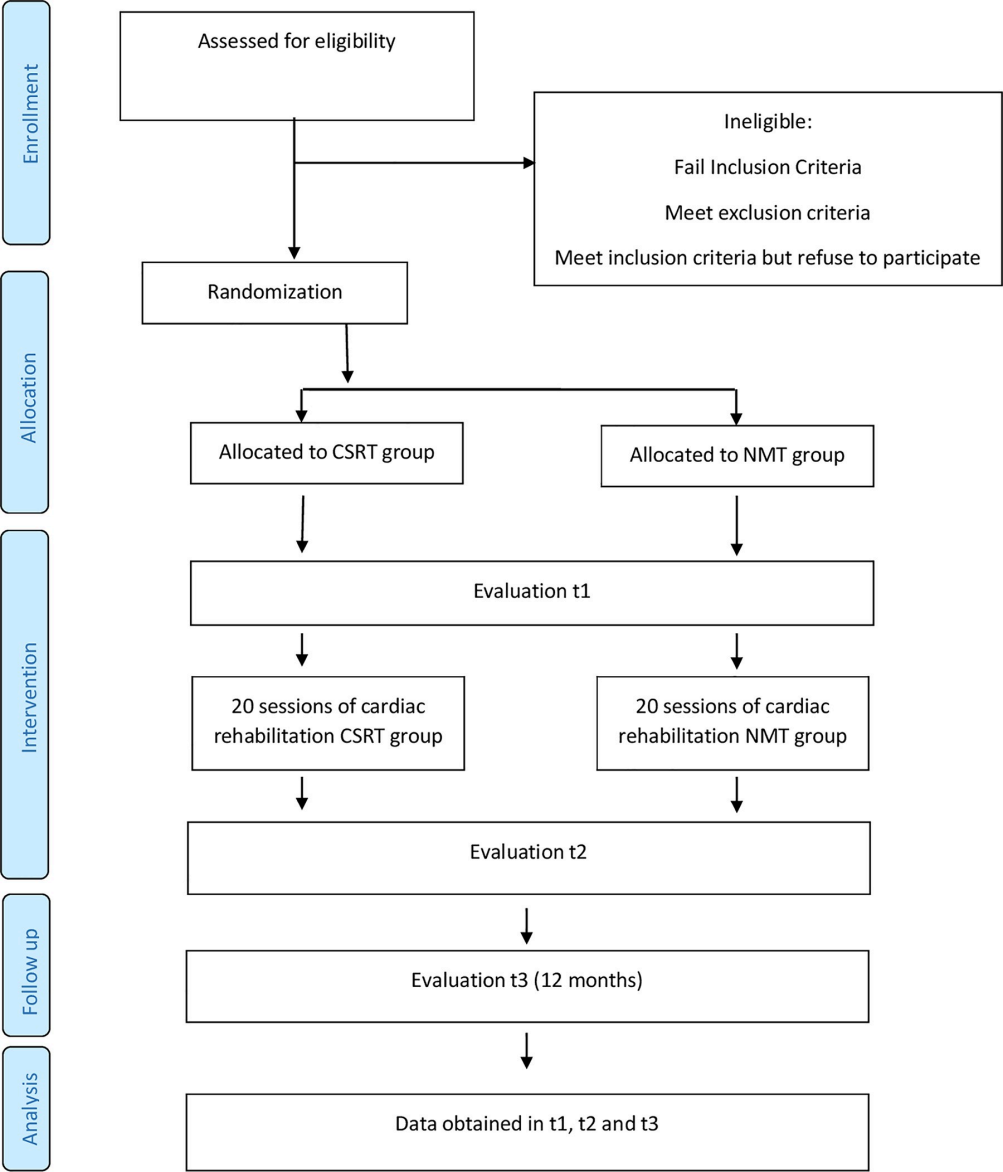
Protocol timeline implemented for the study. CSRT: classic strength-resistance training; NMT: neuromuscular training.

Data will be collected at two evaluation sessions, one before and one at the end of 20 programmed rehabilitation sessions (t1 and t2), as well as at a separate follow-up session scheduled one year later (t3). The study design and progress are outlined in Fig 1.

#### Primary outcome

The primary outcome will be patient performance, according to the Incremental Shuttle Walking Test (ISWT) used to predict VO_2_ max. The ISWT was originally developed to assess patients with chronic obstructive pulmonary disease (COPD) and requires individuals to walk at a gradually increasing speed until they reach a heart rate or symptom limit. The wide range of walking speeds used in the ISWT allows it to accommodate all ambulant patients, from those with a minimal disability to those with more severe symptoms. This test has proven reliability with cardiac rehabilitation patients and correlates well with VO_2_ max and metabolic equivalents as well as with other tests such as the Six-Minute Walking Test [29, 30].

#### Secondary outcomes

Secondary outcomes include patient cardiorespiratory fitness, functional capacity, sexual dysfunction assessment outcomes and quality of life, as assessed using the tests described below.

The Chester Step Test was designed to provide a safe and practical means of assessing aerobic fitness under submaximal conditions; its ability to predict VO_2_ max compared with actual VO_2_ max values ranges from 5% to 15% [31]. The Chester Step Test has been validated for different populations, from healthy adults to patients with COPD and has been used as a risk-factor predictor for cardiovascular disease in several studies [32, 33].

The 30-Second Chair Stand Test is a chair-stand test which focuses on a standardised protocol time rather than a number of repetitions; it allows wide variations in ability levels to be assessed from scores ranging from 0 stands to more than 20 for very fit individuals. This test has been widely used in several populations with frailty or chronic diseases in order to screen for sarcopenia and lower-limb functional capacity [24, 34].

The Single-Leg Squat Test is a closed-chain lower-limb movement used to highlight uncontrolled knee valgus, a phenomenon which could be related to altered hip-knee or ankle kinematics and can lead to injury. 2D analysis is used to screen lower-limb mechanics [35]. This test is also useful for evaluating movement processes for individuals with non-arthritic hip pain [36] Finally, there is a difference in the performance outcomes between active and non-active patients [37], and so this test can be a useful and low-cost way to assess outcomes after having completed an exercise programme.

We will also measure hip flexor strength with a handheld dynamometer, a reliable and validated metric which has been used in several different populations [38]. Because the standardisation of isometric strength values according to body weight has been proven as feasible, this test allows normal patterns to be established and for the impact of injuries to be assessed in terms of muscle strength loss [39].

The Sexual Health Inventory for Men, validated as a 5-item self-administrated questionnaire will also be used in this study. The Sexual Health Inventory for Men is an abbreviated version of the International Index of Erectile Function Test and measures erectile dysfunction in men [40].

The EQ-5D-5L measures HRQoL using the EuroQol Visual Analog Scale and via questions in five areas in a self-administered questionnaire. The EQ-5D-5L is commonly used to measure HRQoL after cardiac surgery and rehabilitation, and has even been used in health cost-analysis studies [41, 42].

#### Safety considerations

All measurements, evaluations, and interventions in the context of the present study will be performed in a safe environment with an emergency resuscitation trolley equipped with a defibrillator, manual ventilation devices, and CPR medications needed. Patient’s vitals will be continuously monitored during measurements and rehabilitation. All adverse events will be evaluated, recorded and discussed in the final paper.

#### Sample size calculation and statistical analysis

The sample size was calculated using G*Power software (version 3.1.9.2) based on our previous pilot study (F tests, ANOVA: Repeated measures, within-between interaction). Considering an effect size of 0.58 (Cohen’s *d*) for the primary outcome (the ISWT result) and assuming a possible 10% dropout rate, a total of 30 patients (15 in each group) will be required to reach a 5% significance level with a power of 90%.

The statistical analysis will be carried out by taking an intention-to-treat approach and the baseline differences between groups will be tested using chi-square and Mann–Whitney U tests to ensure successful randomisation. Missing data will be handled using the last observation carried forward imputation method.

An ANOVA mixed factorial model will be employed to compare the effects of the interventions in each group in terms of cardiorespiratory fitness, functional variables, sexual dysfunction questionnaire and quality of life results, using time (pre-treatment, post-treatment, and the 1-year follow up) as the within-group factor, and the intervention type (NMT or CRST) as the between-group factor. A confidence interval of 95% will be used to establish any differences, and statistical significance will be reported for all between-group differences at *p* < 0.05 (2-sided). If assumption of normality is not being fulfilled, pairwise comparisons between groups will be performed using the alternative non-parametric test, as appropriate, for independent samples (U-Mann-Whitney test) or dependent samples (Wilcoxon test). To avoid error type 1 the alpha level will be adjusted to 0.0033 (Bonferroni Adjustment). All the statistical analyses will be performed using IBM SPSS for Windows (version 24.0, Armonk, NY. IBM. Corp.).

#### Data management

All data will be entered into the database using unique study codes for each participant and will be securely stored on password-protected computer. Data manager, who is independent from competing interests, can only access to the data. A Data Monitoring Committee is not needed since the study is minimal risk. Important protocol modifications during this study will be communicated to the trial registry and the journal of publication.

## Discussion

To the best of our knowledge, this will be the first randomised clinical trial to explore the efficacy of a NMT CRP compared to a CSRT protocol in patients with ACS. CRPs are recommended for these patients in order to improve their cardiorespiratory parameters and have proven beneficial in patients recovering from a cardiac event [20–23]. However, due to the design of our study the results of this randomised clinical trial will be cautiously analysed taking into account the characteristics of the sample involved. Therefore, future studies are paramount to potentially confirm and expand our results to a larger and different population groups. Finally, further research is still required to define which exercise types most efficiently improve both cardiorespiratory fitness and the functional capacities of patients in their everyday lives, that have a high influence in their HRQoL.

Patients with ACS must maintain an active lifestyle to avoid risk-factors and recurrences of cardiac problems. Neuromuscular exercise has been proven as a useful means to decrease the incidence of osteoarticular problems and prevent patients from returning to sedentary lifestyles and the risks associated with inactivity [36, 43]. Furthermore, patients may find neuromuscular workout routines more motivating and engaging, thus encouraging them to adopt healthier lifestyle patterns.

The results from this study will help improve the design of rehabilitation sessions so that they consider cardiorespiratory parameters as well as factors associated with the performance of everyday activities and the sexual health status of patients affected by ACS.

## Supporting information

S1 Checklist SPIRIT checklist. (DOC)

S1 Table Pilot study data. (DOCX)

S1 Protocol Trial protocol study_Spanish versión. (DOCX)

S2 Protocol Trial protocol study_English versión. (DOCX)
