# Supplementary material for: Effects of neuromuscular training compared to classic strength-resistance training in patients with acute coronary syndrome: A study protocol for a randomized controlled trial
Source: PLoS One. 2020 Dec 23;15(12):e0243917. doi: 10.1371/journal.pone.0243917 (PMC7757882; doi:10.1371/journal.pone.0243917)
Supplement: S1 Table — (DOCX) [file pone.0243917.s002.docx]

S1A Table. Descriptive characteristics of the pilot study sample

| **Parameter** | **NMT (n=5)** | **CSRT (n=5)** |
| --- | --- | --- |
| **Gender** | 5 ♂ | 4 ♂ / 1 ♀ |
| **Age (years)** | 56,2 (7,3) | 62,6 (14,0) |
| **Height (cm)** | 170,2 (2,9) | 165,7 (6,2) |
| **Weight (kg)** | 82,9 (12,3) | 84,4 (19,4) |
| **BMI (kg/m^2^)** | 28,6 (4,3) | 30,5 (6,1) |
| **BFP (%)** | 25,4 (2,7) | 31,9 (5,1) |
| **EQ-5D-5L scores**  Mobility (% with problems)  Self-care (% with problems)  Usual activities (% with problems)  Pain/disconfort (% with problems)  Anxiety/depression (% with problems) | 0% 0% 20% 40% 80% | 20% 0% 40% 20% 40% |

Data are mean (SD).

♂ male; ♀ female; BFP body fat percentage; BMI body mass index; CSRT classic strength-resistance training; NMT neuromuscular training

S1B Table. Intragroup and between groups differences of the outcome measures of the pilot study

| **OUTCOME** | **NMT** | | **CSRT** | | **INTRA-GROUP DIFFERENCES (T2 – T1)** | | **BETWEEN-GROUP DIFFERENCES (NMT – CSRT )** |
| --- | --- | --- | --- | --- | --- | --- | --- |
|  | **T1** | **T2** | **T1** | **T2** | **NMT** | **CSRT** | **T2** |
| **Incremental Shuttle Walking Test (m)** | 623,3 [299,2 947,3] | 725,6 [415,4 1035,8] | 458,7 [134,7 782,8] | 530,4 [220,2 840,7] | 102,3 [12,7 191,9]***** | 71,7 [-17,9 161,3] | 195,2 [-259,8 650,1] |
| **Chester Step Test (s)** | 451,8 [249,9 653,8] | 571,2 [439 703,3] | 318,4 [116,4 520,3] | 386 [253,9 518,2] | 119,3 [7,6 231]***** | 67,7 [-44 179,4] | 185,1 [-8,7 378,9] |
| **30-second Chair Stand Test** | 13,2 [8,6 17,8] | 17,2 [12,4 22] | 11,8 [7,2 16,4] | 13,2 [8,4 18] | 4 [2 5,9]***** | 1,4 [-0,5 3,3] | 3,9 [-3,1 11] |
| **Hip Flexor Handled Dynamometry (R)** | 269,8 [179,2 360,4] | 315,2 [240,2 390,2] | 279,4 [188,8 370] | 269 [194 344] | 45,4 [11,4 79,5]***** | -10,4 [-44,4 23,7] | 46,2 [-63,8 156,1] |
| **Hip Flexor Handled Dynamometry (L)** | 258,9 [177,4 340,5] | 305,9 [249 362,7] | 258,2 [176,7 339,7] | 245,6 [188,8 302,5] | 46,9 [3,7 90,1]***** | -12,5 [-55,8 30,7] | 60,2 [-23,2 143,6] |
| **Sexual Health Inventory for Men (1-25)** | 12,5 [4,5 26,5] | 15,5 [4,5 26,5] | 14,1 [1,7 26,3] | 14,7 [3,7 25,7] | 2,9 [0,1 0,5] ***** | 0,6 [0,5 -1,7] | 0,8 [0,9 -15,3] |
| **EQ­_index value** | 0,7 [0,4 0,9] | 0,8 [0,6 1] | 0,9 [0,7 1,1] | 0,9 [0,8 1,1] | 0,1 [-0,8 0,3] | 0,04 [-1,3 0,2] | -0,1 [-0,4 0,1] |
| **EQ­_VAS (0-100)** | 60,7 [32,9 88,5] | 69,8 [51,2 88,5] | 76,4 [48,7 104,2] | 83,3 [64,7 102,1] | 9,1 [-3,1 21,4] | 6,9 [-5,4 19,1] | -13,5 [-40,9 13,9] |
| **QLPAMI (44-220)** | 94,7 [58,2 131,3] | 90,1 [52,4 127,8] | 79,2 [42,7 115,8] | 81,5 [43,7 119,2] | -4,6 [-10,3 1,1] | 2,2 [-3,5 7,9] | 8,6 [-46,7 64] |

Data are mean [IC 95%].

CSRT: classic strength-resistance training; NMT: neuromuscular training; EQ_index value: EuroQol index value; EQ_VAS: EuroQol visual analogue scale; QLPAMI: Quality of life post acute myocardial infarction; L: left;, R: right; T1: before intervention; T2: after intervention; ******P-value*< 0,05
