# Supplementary material for: Effects of neuromuscular training compared to classic strength-resistance training in patients with acute coronary syndrome: A study protocol for a randomized controlled trial
Source: PLoS One. 2020 Dec 23;15(12):e0243917. doi: 10.1371/journal.pone.0243917 (PMC7757882; doi:10.1371/journal.pone.0243917)
Supplement: S1 Protocol — (DOCX) [file pone.0243917.s003.docx]

**EFECTO DEL ENTRENAMIENTO NEUROMUSCULAR SOBRE LAS CAPACIDADES FUNCIONALES EN PACIENTES TRAS INFARTO AGUDO DE MIOCARDIO**

**INTRODUCCIÓN**

El **infarto agudo de miocardio (IAM)** se define como un cuadro clínico producido por la isquemia y posterior muerte de una porción más o menos extensa de miocardio, consecuencia de la obstrucción de una arteria coronaria. Cuando se produce la obstrucción al flujo, se suprime el aporte sanguíneo al tejido miocárdico, produciendo un déficit de oxígeno que, si se mantiene en el tiempo, produce la necrosis no reversible de la zona miocárdica afectada^1,2^.

En la pasada década, la cardiopatía isquémica provocó hasta un 29% de los fallecimientos en países industrializados convirtiéndose en la principal causa de muerte, dato que se mantendrá en 2020 según las últimas proyecciones^3^. Sin embargo, se observa una disminución de la mortalidad por cardiopatía isquémica, relacionada con una menor incidencia y con las mejoras introducidas en los últimos años tanto en el tratamiento en fase aguda del infarto agudo de miocardio como en la prevención primaria ^4,5^.

Las **manifestaciones clínicas** de la cardiopatía son amplias, con el dolor torácico como principal signo, presentándose con fuerte intensidad y sin ceder ante el reposo. La irradiación de dicho dolor puede ser variable (interescapular, mandibular, cervical, miembros superiores, etc.) y puede acompañarse de signos inespecíficos como la disnea, el sudor y los mareos, entre otros^1,6^.

Además de la sintomatología aguda propia de la patología, después de un IAM los pacientes tienen que aprender a vivir con las consecuencias de la enfermedad y su tratamiento, lo que puede conllevar un deterioro de la **calidad de vida relacionada con la salud** (CVRS)^7,8^. No existe consenso sobre el concepto de CVRS pero en lo que coinciden los distintos autores es en que se trata de un concepto complejo y multidimensional que adquiere una gran importancia a la hora de evaluar el impacto de una enfermedad o problema de salud, en cuyo caso hablamos de CVRS. Este concepto engloba aspectos tales como los síntomas físicos; la función cognitiva y bienestar psicológico; el rendimiento o rol y bienestar social; el estado global de salud; la percepción del cuidado y constructos personales^8,9^.

La **actividad sexual** es un aspecto que afecta a la CVRS de los pacientes con infarto agudo de miocardio (IAM) y sus cónyuges^10,11^. Tanto el tratamiento habitual de los pacientes, como las adaptaciones cardiovasculares durante el acto sexual, pueden agravar la sintomatología del cardiópata al aumentar el consumo de oxígeno^12-14^. Este consumo energético durante el acto sexual en la posición habitual y con el compañero habitual equivale a 3-4 METs (Equivalente metabólico o *metabolic equivalent of task*), siendo un MET el consumo de oxigeno por minuto de una persona en reposo en relación con su peso corporal. Aunque la actividad sexual es un factor de riesgo para pacientes que han sufrido IAM, los pacientes que pueden hacer ejercicio cuyo consumo energético es equivalente o superior a 5 METS, están en disposición de reanudar la actividad sexual^12,15^.

La importancia de un estilo de vida cardiosaludable, que incluya la realización de ejercicio físico, ha sido descrita y evidenciada tanto en población adulta como pediátrica^16^. La implementación de programas de evaluación integral de la función cardiopulmonar y la respuesta a la actividad física, así como el diseño de una rehabilitación cardíaca basada en dicha evaluación, pueden favorecer la calidad de vida de los pacientes **cardiópatas tras infarto agudo de miocardio**, además de resultar de gran utilidad para la evaluación del estado clínico y el pronóstico del paciente.

Los **programas de rehabilitación cardíaca** (PRC) son sistemas de *actuación multifactorial* recomendados por la Organización Mundial de la Salud desde la década de los sesenta. Existe evidencia científica de que dichos programas no sólo favorecen el control de los factores de riesgo cardiovascular (FRCV), sino que actúan sobre la calidad de vida, favoreciendo la reinserción laboral y disminuyendo la morbi-mortalidad de los pacientes, además de demostrar de forma inequívoca su viabilidad al haberse constatado su eficacia a nivel de coste-beneficio^17-19^.

Las *contraindicaciones* para efectuar los PRC se han visto reducidas en los últimos años gracias al mejor conocimiento de los resultados y peligros inherentes a la práctica del ejercicio. Aquellas que podrían considerarse como absolutas se reducen a los aneurismas disecantes de aorta y obstrucciones severas del tracto de salida del ventrículo izquierdo sin indicación quirúrgica^1,4^.

Los PRC se dividen en tres *fases*. La Fase I comprende la estancia hospitalaria, que puede darse también con un paciente en fase previa y posterior a cirugía cardiovascular. Esta fase comprende la movilización precoz y los ejercicios respiratorios, además del control de los FRCV y la actuación psicológica. La fase II tiene una duración aproximada de dos meses y se inicia desde el momento del alta hospitalaria. Los datos clínicos y resultantes de las exploraciones (como son ecocardiograma, ergometría, medicina nuclear, etc.) permiten clasificar a los pacientes en diferentes niveles de riesgo. Esta fase consta de 20-24 sesiones con dos o tres sesiones presenciales en el centro de rehabilitación, que incluyen un período de 10 minutos de calentamiento, un entrenamiento aeróbico de 30-40 minutos y una fase de recuperación de 10 minutos. La intensidad del ejercicio se determina según los datos de una prueba de esfuerzo, y se incrementa en función de la respuesta cronotrópica y tensional del paciente al entrenamiento, además de su percepción subjetiva de esfuerzo. La fase III se mantiene el resto de la vida del paciente. Se realiza de forma extrahospitalaria y, tras un informe escrito del cardiólogo, el paciente cardiovascular continúa con las pautas de ejercicio físico marcadas, el control de los FRCV y los aspectos socio-laborales y psicológicos en centros dotados del equipo material y humano necesario para la continuación de su rehabilitación^1,4,16,18,20^.

En los últimos años el estudio de la disfunción del movimiento y su influencia en la eficiencia del rendimiento del gesto deportivo, en especial del miembro inferior, ha tenido un desarrollo considerable. Desde los estudios de *Sahrmann et al.*^21-25^, hasta los más recientes realizados por *Comerford et al.*^26,27^, se ha evidenciado la importancia del **control neuromuscular** para mejorar la calidad del movimiento y prevenir las lesiones derivadas de esta falta de control. Este tipo de entrenamiento se centra en varios elementos clave como la estabilidad del core, realizarlos en una postura funcional, trabajos de fuerza en miembro inferior y ejercicios funcionales^28^.

A pesar de que el grueso de la evidencia sobre el beneficio de este tipo de trabajo es en el mundo del deporte por su importancia para prevenir lesiones y mejorar el control y la propiocepción de los segmentos articulares, otros estudios están probando la efectividad de este trabajo para otras poblaciones^29,30^. Por el ejemplo, el programa GLA:DTM, un programa de **entrenamiento neuromuscular** dirigido por fisioterapeutas a lo largo de toda Dinamarca se está llevando a cabo para mejorar la sintomatología causada por la osteoartritis de cadera en población adulta y anciana. Sin embargo, sus autores apuntan la necesidad de estudiar el potencial de este tipo de trabajo no sólo en patologías de etiología neuromuscular, sino en otras enfermedades crónicas o sobrevenidas y en la capacidad de mejorar el estado general del paciente^31^. Por este motivo, proponemos este estudio para conocer los posibles beneficios de este trabajo respecto a un protocolo tradicional de fuerza.

**OBJETIVOS**

El **objetivo general** del estudio se basa en comprobar si el entrenamiento neuromuscular es más efectivo respecto al entrenamiento de fuerza clásico de los programas de Rehabilitación Cardíaca para mejorar las capacidades funcionales de los pacientes tras infarto agudo de miocardio.

Los **objetivos específicos** del presente proyecto son:

- Analizar el efecto de la intervención sobre parámetros funcionales de miembros inferiores

- Determinar el efecto del programa sobre variables de fuerza de musculatura respiratoria y periférica

- Analizar el impacto de un programa de rehabilitación cardíaca en la calidad de vida de los pacientes que han sufrido un IAM

- Determinar las variables que influyen en la actividad sexual de los pacientes tras IAM y el impacto en su calidad de vida

**METODOLOGÍA**

**Diseño**

El estudio se plantea como un **ensayo controlado aleatorizado**, procedimiento científico experimental caracterizado por la distribución aleatoria de los miembros de un grupo de población idónea en un grupo intervención y un grupo control, comparando un impacto específico entre los grupos tras un período establecido de antemano.

El diseño del mismo es un **pre-post**, basándose en la medición y comparación de las variables a estudiar antes y después de la exposición de los participantes a la intervención experimental.

**Participantes**

Los participantes serán seleccionados atendiendo a los siguientes criterios:

Criterios de Inclusión:

 Pacientes mayores de edad diagnosticados de síndrome coronario agudo con o sin elevación del segmento ST (SCASEST / SCACEST), con una estratificación de riesgo Bajo o Medio según los tests de esfuerzo realizados por servicio de cardiología del Hospital IMED Burjassot.

 Prescripción médica.

 Consentimiento firmado por parte del paciente *(Anexo I)*.

Criterios de Exclusión:

 Patologías que contraindiquen el ejercicio: enfermedad inflamatoria cardíaca en fase aguda, disfunción severa o moderada de VI o VD, IC descompensada, arritmias graves documentadas en DAI, enfermedad coronaria significativa, HTAP >40mmHg o que requiera tratamiento con vasodilatadores, estenosis aórtica con gradiente sistólico en reposo >50mmHg, estenosis pulmonar con gradiente sistólico en reposo <50mmHg, hipertensión sistémica >95% percentil, Sat.O2 basal <90%, insuficiencia severa de válvula AV, enfermedad renal grave en fase aguda, hepatitis aguda e infección activa.

 Alteraciones en la ergoespirometría: arritmias graves inducidas por el ejercicio, depresión ST con esfuerzo, respuesta hipertensiva exagerada, hipotensión al esfuerzo y dolor torácico.

**Intervención: programa de entrenamiento**

La fase II del programa de rehabilitación cardíaca estará basada en el modelo F.I.T.T.^32,33^, donde los parámetros destacados son la frecuencia de entrenamiento, la intensidad del ejercicio, la duración de las sesiones/programa completo, y el tipo de ejercicio planteado:

- FREQUENCY. 2 sesiones por semana en gimnasio de rehabilitación cardíaca. Además, el trabajo se complementará con una pauta de ejercicio domiciliario.

- INTENSITY. Intensidad basada en el consumo de oxígeno (VO2) máximo y la frecuencia cardíaca (FC) máxima, calculada en base a las pruebas ergoespirométricas realizadas. La FC de Entrenamiento (FCE) será aquella dada por los umbrales ventilatorios obtenidos en las curvas de Wasserman.

- TIME. Las sesiones tendrán una duración aproximada de 60 minutos (aproximadamente 40 minutos de ellos correspondían a la fase de entrenamiento). El programa incluirá un total de 20 sesiones (2 sesiones a la semana durante 10 semanas aproximadamente).

- TYPE. El tipo de trabajo indicado en los programas de rehabilitación cardíaca en la fase aeróbica será la modalidad contínua e interválica, que se establecerá dependiendo de la estratificación de riesgo del paciente.

Todos los pacientes se encontrarán monitorizados a través de un dispositivo de reconocimiento de la frecuencia cardíaca y saturación de O2. Además, se controlará en todo momento la percepción subjetiva de esfuerzo del paciente mediante la Escala de Borg modificada, tanto a nivel de disnea como de fatiga de miembros inferiores.

Las sesiones se estructurarán de la siguiente forma:

**Fase de Calentamiento (10 minutos)**:

- Ejercicios respiratorios: Diafragmáticos + entrenamiento de la musculatura respiratoria

- Marcha suave

**Fase de Entrenamiento (40 minutos):**

- Fase Ejercicio Aeróbico en tapiz rodante / cicloergómetro (20 minutos)

- Fase Ejercicio Fuerza (20 minutos) *(Anexo II)*

**Fase de Enfriamiento (10 minutos):**

- Marcha suave

- Estiramientos

**Variables evaluadas**

Variables sociodemográficas: edad, sexo, nivel de estudios, tipo de trabajo, estado civil, tiempo de relación con la pareja.

Variables dependientes e independientes:

La evaluación de dichas variables se realizará en conjunto con el servicio de cardiología del Hospital IMED Valencia. En el Hopsital IMED se realizarán aquellas pruebas relativas a la estratificación de riesgo del paciente (ergoespirometría, ecocardiografía y perfil bioquímico), y en la Universidad CEU Cardenal Herrera se evaluarán las capacidades respiratorias y funcionales del paciente (medición de presiones, test incremental Shuttle, evaluación de la fuerza, parámetros funcionales de miembro inferior), además de los cuestionarios de calidad de vida y disfunción sexual.

Todas las variables se evaluarán en 3 momentos distintos: pre-intervención, post-intervención inmediata y un año posteriormente a la finalización del estudio.

- Servicio de Cardiología (Hospital IMED Valencia):

o **Tolerancia al Esfuerzo,** evaluada mediante ergoespirometría. El test de esfuerzo con consumo de oxígeno (CPET) consiste en someter al paciente a un ejercicio físico con una carga de trabajo controlada y progresiva, realizar medidas antes, durante, y después de la realización de dicho trabajo, y posteriormente analizar los datos obtenidos. Esta prueba nos aporta información sobre su función cardiaca, pulmonar, musculo-esquelética, y la respuesta de todas ellas al ejercicio físico, constituyendo una información esencial para una correcta evaluación de la situación del paciente, establecimiento de un pronóstico, y un buen punto de partida para el diseño de programas de rehabilitación cardiaca en población tras infarto agudo de miocardio. Entre el equipo necesario es imprescindible un cicloergómetro / tapiz rodante, además de equipamiento de medida y análisis, como es una torre de esfuerzo cardiopulmonar, carro metabólico, electrocardiograma, pulsioxímetro, monitor de tensión arterial, y equipamiento adicional (carro de reanimación, camilla de exploración, bala de oxígeno) y accesorios^34^.

o **Fracción de Eyección,** medida mediante ecocardiografía. Se define como fracción de eyección el volumen sistólico dividido por el volumen del ventrículo al final de la diástole. En el ventrículo izquierdo suele tener un valor de unos dos tercios (55 a 65%). En los pacientes que han sufrido un infarto agudo de miocardio, la fracción de eyección sirve como un indicador de riesgo, junto con otros elementos. Así, se puede estratificar el riesgo del paciente en Bajo, Medio y Alto.

o **Bioquímica.** Los marcadores cardíacos se utilizan durante las primeras horas para el diagnóstico y evaluación del daño miocárdico secundario a un IAM. Además de la evaluación de estos marcadores, numerosos parámetros pueden verse alterados ante un paciente con factores de riesgo cardiovascular, como en aquellos con antecedentes de dislipemia o diabetes. El seguimiento estándar de los pacientes incluye análisis bioquímico de niveles de triglicéridos, colesterol total, colesterol HDL y LDL, y hemoglobina glicosilada. En este estudio se incluirán además marcadores de inflamación como proteína C reactiva e interleucina 6, y NT-proBNP en aquellos pacientes con insuficiencia cardíaca asociada. Asimismo, se incluirán parámetros de metabolismo de hierro (hierro sérico, ferritina, índice de saturación de la transferrina, transferrina sérica).

- Servicio de Rehabilitación (Universidad CEU Cardenal Herrera):

o **Incremental Shuttle Walking Test (ISWT).** Es un test derivado del *“20-metre Shuttle run test”* adaptado a los pacientes cardíacos, como aquellos con enfermedad coronaria. El ISWT ha demostrado su efectividad en el ámbito de la rehabilitación cardíaca, además de correlacionarse con el VO2 máximo y los equivalentes metabólicos del paciente cardiovascular. El paciente debe caminar entre dos marcas distanciadas 9 metros, aumentando su velocidad de forma progresiva^35-37^.

o Capacidades funcionales:

 *30’’ stand to sit test.* Mide el máximo número de sentadillas que el paciente es capaz de realizar en 30 segundos. El objetivo es evaluar la resistencia muscular y los cambios rápidos entre contracción concéntrica y excéntrica de la musculatura del miembro inferior. Desde una posición de sedestación, el paciente se incorpora completamente en posición de bipedestación y vuelve a sentarse completamente tantas veces como pueda en el tiempo establecido^38^.

 *Cuarto de sentadilla monopodal.* Se pide al sujeto que realice una flexión de rodilla en apoyo monopodal. El evaluador puntúa la ejecución según lo definido por Comerford y Motram^26,39^.

 *Fuerza de extensores de la cadera.* Se realizará mediante un dinamómetro hand-held, llevando a cabo 3 medidas de una contracción isométrica concéntrica^38,40^.

 *Chester Step Test.* En esta prueba submáxima, el paciente debe subir y bajar a un escalón a ritmo de un metrónomo, aumentando la velocidad progresivamente. La altura del escalón dependerá de la edad y la actividad física previa del sujeto. Este test ha demostrado ser un buen indicador de los factores de riesgo cardiovascular y predictivo del riesgo cardiovascular^41,42^.

o **Presión inspiratoria estática máxima** (PIM, cmH2O) / **Presión espiratoria estática máxima** (PEM, cmH2O), o presión positiva y negativa máxima, respectivamente, que puede generar y mantener el conjunto de la musculatura respiratoria, contra un circuito ocluido, durante al menos un 1 segundo^43^. Se determina a nivel o cerca de la capacidad pulmonar total y el volumen residual^44^. Se realizan un mínimo de 3 mediciones, separadas de 1 minuto, y para los resultados se selecciona el mayor valor de los 3 extraídos. Se utilizará el dispositivo MicroRPM *(Carefusion, U.K.).*

- Servicio de Enfermería (Universidad CEU Cardenal Herrera):

o **Calidad de vida y disfunción sexual.** Para la valoración de estos parámetros se utilizarán diferentes cuestionarios:

 *EuroQol (EQ-5D).* Consta de dos partes. La primera corresponde a la descripción del estado de salud en cinco dimensiones (movilidad, cuidado personal, actividades cotidianas, dolor/malestar y ansiedad/depresión). En la segunda parte del cuestionario el sujeto debe puntuar sobre una escala visual analógica milimetrada de su estado de salud en el momento que se autoclasifica o autopercepciona su estado personal, teniendo en los extremos de la escala el peor estado de salud (0) y el mejor estado de salud (100)^45^.

 El cuestionario auto-administrado *Sexual Health Inventory for Men,* validado y consistente en 5 ítems, también será utilizada en este estudio. Este cuestionario es una versión abreviada del International Index of Erectile Function Test y evalúa la disfunción eréctil en hombres^46^*.*

**ANÁLISIS ESTADÍSTICO**

El análisis de los datos se realizará con el programa estadístico IBM SPSS for Windows, versión 24.0. Armonk, NY: IBM Corp.

El cálculo del tamaño muestral se realizará en base a los resultados en un estudio piloto previo.

La comparación de los resultados obtenidos entre el grupo Intervención y el grupo Control antes y después de la intervención se realizará mediante un modelo factorial mixto (Split-plot). El factor intra-sujetos será el tiempo (PRE, POST, POST1). El factor inter-sujetos será el grupo, con dos niveles: Intervención y Control.

Se considerará un intervalo de confianza de 95% para la diferencia y la significatividad estadística se establecerá con una p<0.05.

**BIBLIOGRAFÍA**

(1) Velasco JA, Cosín J, Maroto JM, Muñiz J, Casasnovas JA, Plaza I, et al. Guías de práctica clínica de la Sociedad Española de Cardiología en prevención cardiovascular y rehabilitación cardíaca. Revista Española de Cardiología 2000;53(8):1095-1120.

(2) Maroto, JM. De Pablo, C. Artigao, R. Rehabilitación Cardíaca. Sociedad Española de Cardiología. Olalla Cardiología Ediciones 2009:500-549.

(3) Maroto Montero JM, Artigao Ramírez R, Morales Durán MD, de Pablo Zarzosa C, Abraira V. Rehabilitación cardíaca en pacientes con infarto de miocardio. Resultados tras 10 años de seguimiento. Revista española de cardiología 2005;58(10):1181-1187.

(4) de la Cuerda, Roberto Cano, Diego IMA, Martín JJA, Sánchez AM, Page JCM. Programas de rehabilitación cardiaca y calidad de vida relacionada con la salud. Situación actual. Revista Española de Cardiología 2012;65(1):72-79.

(5) Wood DA. Clinical reality of coronary prevention guidelines: a comparison of EUROASPIRE I and II in nine countries. The Lancet 2001;357(9261):995-1001.

(6) Kassab Y, Hassan Y, Aziz NA, Ismail O, AbdulRazzaq H. Patients’ adherence to secondary prevention pharmacotherapy after acute coronary syndromes. International journal of clinical pharmacy 2013;35(2):275-280.

(7) Medrano MJ, Cerrato E, Boix R, Delgado-Rodríguez M. Factores de riesgo cardiovascular en la población española: metaanálisis de estudios transversales. Medicina clínica 2005;124(16):606-612.

(8) Grau M, Elosua R, de Leon AC, Guembe MJ, Baena-Díez JM, Alonso TV, et al. Factores de riesgo cardiovascular en España en la primera década del siglo XXI: análisis agrupado con datos individuales de 11 estudios de base poblacional, estudio DARIOS. Revista Española de Cardiología 2011;64(4):295-304.

(9) Bauer LK, Caro MA, Beach SR, Mastromauro CA, Lenihan E, Januzzi JL, et al. Effects of depression and anxiety improvement on adherence to medication and health behaviors in recently hospitalized cardiac patients. Am J Cardiol 2012;109(9):1266-1271.

(10) Thylén I, Brännström M. Intimate relationships and sexual function in partnered patients in the year before and one year after a myocardial infarction: a longitudinal study. European Journal of Cardiovascular Nursing 2015;14(6):468-477.

(11) Rosman L, Cahill JM, McCammon SL, Sears SF. Sexual health concerns in patients with cardiovascular disease. Circulation 2014 Feb 4;129(5):e313-6.

(12) Levine GN, Steinke EE, Bakaeen FG, Bozkurt B, Cheitlin MD, Conti JB, et al. Sexual activity and cardiovascular disease: a scientific statement from the American Heart Association. Circulation 2012 Feb 28;125(8):1058-1072.

(13) Lim S, Sim Ds, Han J. The factors associated with sexual recovery in male patients with acute myocardial infarction under phase II cardiac rehabilitation. J Clin Nurs 2016;25(19-20):2827-2834.

(14) Bispo GS, de Lima Lopes J, de Barros AL. Cardiovascular changes resulting from sexual activity and sexual dysfunction after myocardial infarction: integrative review. J Clin Nurs 2013;22(23-24):3522-3531.

(15) Dahabreh IJ, Paulus JK. Association of episodic physical and sexual activity with triggering of acute cardiac events: systematic review and meta-analysis. JAMA 2011;305(12):1225-1233.

(16) Espinosa J, De Teresa C, Navas J. Rehabilitación en afecciones cardíacas. Afecciones médicas en fisioterapia. 1ª ed. Málaga: Spicum; 1999. p. 467-477.

(17) Amigo Castañeda P, Amigo González R, Rodríguez Díaz M, Castañeda Gueimonde CM. Modificación de algunos factores de riesgo coronario después de la rehabilitación física. Revista Médica Electrónica 2010;32(3):0-0.

(18) Caliani JSE, Navas JCB. La rehabilitación cardiaca en el centro de salud. Intervención del fisioterapeuta. Rehabilitación cardíaca y atención primaria. 2ª ed. Madrid: Ed. Médica Panamericana; 2002. p. 95-112.

(19) Plaza Pérez I. Estado actual de los programas de prevención secundaria y rehabilitación cardiaca en España. Revista Española de Cardiología 2003;56(08):757-760.

(20) O’Donnell CJ, Elosua R. Factores de riesgo cardiovascular. Perspectivas derivadas del Framingham Heart Study. Revista española de Cardiología 2008;61(3):299-310.

(21) Van Dillen LR, Sahrmann SA, Norton BJ, Caldwell CA, Fleming D, McDonnell MK, et al. Effect of active limb movements on symptoms in patients with low back pain. Journal of Orthopaedic & Sports Physical Therapy 2001;31(8):402-418.

(22) Van Dillen LR, McDonnell MK, Fleming DA, Sahrmann SA. Effect of knee and hip position on hip extension range of motion in individuals with and without low back pain. Journal of Orthopaedic & Sports Physical Therapy 2000;30(6):307-316.

(23) Van Dillen LR, Maluf KS, Sahrmann SA. Further examination of modifying patient-preferred movement and alignment strategies in patients with low back pain during symptomatic tests. Man Ther 2009;14(1):52-60.

(24) Van Dillen LR, Sahrmann SA, Norton BJ, Caldwell CA, McDonnell MK, Bloom NJ. Movement system impairment-based categories for low back pain: stage 1 validation. Journal of Orthopaedic & Sports Physical Therapy 2003;33(3):126-142.

(25) Van Dillen LR, Sahrmann SA, Norton BJ, Caldwell CA, Fleming DA, McDonnell MK, et al. Reliability of physical examination items used for classification of patients with low back pain. Phys Ther 1998 Sep;78(9):979-988.

(26) Comerford M, Mottram S. Kinetic control: the management of uncontrolled movement. : Elsevier Australia; 2012.

(27) Comerford MJ, Mottram SL. Movement and stability dysfunction–contemporary developments. Man Ther 2001;6(1):15-26.

(28) Ageberg E, Link A, Roos EM. Feasibility of neuromuscular training in patients with severe hip or knee OA: the individualized goal-based NEMEX-TJR training program. BMC musculoskeletal disorders 2010;11(1):126.

(29) Ageberg E, Nilsdotter A, Kosek E, Roos EM. Effects of neuromuscular training (NEMEX-TJR) on patient-reported outcomes and physical function in severe primary hip or knee osteoarthritis: a controlled before-and-after study. BMC musculoskeletal disorders 2013;14(1):232.

(30) Steib S, Rahlf AL, Pfeifer K, Zech A. Dose-Response Relationship of Neuromuscular Training for Injury Prevention in Youth Athletes: A Meta-Analysis. Frontiers in physiology 2017;8:920.

(31) Skou ST, Roos EM. Good Life with osteoArthritis in Denmark (GLA: D™): evidence-based education and supervised neuromuscular exercise delivered by certified physiotherapists nationwide. BMC musculoskeletal disorders 2017;18(1):72.

(32) American College of Sports Medicine. ACSM's guidelines for exercise testing and prescription. : Lippincott Williams & Wilkins; 2013.

(33) Billinger SA, Boyne P, Coughenour E, Dunning K, Mattlage A. Does aerobic exercise and the FITT principle fit into stroke recovery? Current neurology and neuroscience reports 2015;15(2):519.

(34) Wasserman K, Hansen JE, Sue DY, Stringer WW, Whipp BJ. Principles of exercise testing and interpretation: including pathophysiology and clinical applications. Medicine & Science in Sports & Exercise 2005;37(7):1249.

(35) Casillas J, Hannequin A, Besson D, Bénaïm S, Krawcow C, Laurent Y, et al. Walking tests during the exercise training: specific use for the cardiac rehabilitation. Annals of physical and rehabilitation medicine 2013;56(7-8):561-575.

(36) Hanson LC, Taylor NF, McBurney H. The 10 m incremental shuttle walk test is a highly reliable field exercise test for patients referred to cardiac rehabilitation: a retest reliability study. Physiotherapy 2016;102(3):243-248.

(37) Pichurko BM. Exercising your patient: which test(s) and when? Respir Care 2012 Jan;57(1):100-10; discussion 110-3.

(38) Dobson F, Hinman R, Roos EM, Abbott J, Stratford P, Davis A, et al. OARSI recommended performance-based tests to assess physical function in people diagnosed with hip or knee osteoarthritis. Osteoarthritis and cartilage 2013;21(8):1042-1052.

(39) Weeks BK, Carty CP, Horan SA. Kinematic predictors of single-leg squat performance: a comparison of experienced physiotherapists and student physiotherapists. BMC musculoskeletal disorders 2012;13(1):207.

(40) Andrews AW, Thomas MW, Bohannon RW. Normative values for isometric muscle force measurements obtained with hand-held dynamometers. Phys Ther 1996;76(3):248-259.

(41) Buckley JP, Sim J, Eston RG, Hession R, Fox R. Reliability and validity of measures taken during the Chester step test to predict aerobic power and to prescribe aerobic exercise. Br J Sports Med 2004 Apr;38(2):197-205.

(42) Gray BJ, Stephens JW, Williams SP, Davies CA, Turner D, Bracken RM, et al. Cardiorespiratory fitness is a stronger indicator of cardiometabolic risk factors and risk prediction than self-reported physical activity levels. Diabetes and Vascular Disease Research 2015;12(6):428-435.

(43) Black LF, Hyatt RE. Maximal respiratory pressures: normal values and relationship to age and sex. Am Rev Respir Dis 1969;99(5):696-702.

(44) American Thoracic Society/European Respiratory Society. ATS/ERS Statement on respiratory muscle testing. Am J Respir Crit Care Med 2002 Aug 15;166(4):518-624.

(45) Herdman M, Badía X, Berra S. El EuroQol-5D: una alternativa sencilla para la medición de la calidad de vida relacionada con la salud en atención primaria. Atención primaria 2001;28(6):425-429.

(46) Cappelleri JC, Siegel RL, Glasser DB, Osterloh IH, Rosen RC. Relationship between patient self-assessment of erectile dysfunction and the sexual health inventory for men. Clin Ther. 2001;23(10):1707–19.

**ANEXO I DOCUMENTO DE CONSENTIMIENTO PARA LA PARTICIPACIÓN EN UN PROYECTO DE INVESTIGACIÓN**

Efecto del entrenamiento neuromuscular sobre las capacidades funcionales en pacientes tras infarto agudo de miocardio / IP del proyecto: Noemí Valtueña Gimeno

**D./ Dña._______________________________________________________**

**Con DNI Nº____________________**

**Libre y voluntariamente**

**MANIFIESTO:**

**1.** He leído y comprendido la hoja informativa objeto del estudio.

2. He tenido la oportunidad de hacer preguntas.

3. Mis preguntas han sido respondidas de forma satisfactoria.

4. He recibido información suficiente del estudio y de las pruebas a realizar.

5. Entiendo que la participación es voluntaria y puedo abandonar el estudio cuando lo desee sin que tenga que dar explicaciones y sin que ello afecte a mis cuidados médicos.

6. De acuerdo con lo establecido por el Reglamento (UE) 2016/679 del Parlamento Europeo y del Consejo, de 27 de abril de 2016, relativo a la protección de las personas físicas en lo que respecta al tratamiento de datos personales y a la libre circulación de estos datos y por el que se deroga la Directiva 95/46/CE, he sido informado de que mis datos personales, obtenidos mediante la cumplimentación de este formulario así como los resultantes de mi participación en el proyecto van a ser tratados bajo la responsabilidad de la FUNDACIÓN UNIVERSITARIA SAN PABLO CEU (en adelante, FUSP-CEU), con la finalidad de gestionar mi participación en el presente proyecto de investigación. Además, he sido informado de los siguientes aspectos:

a. Que está prevista la elaboración de perfiles al objeto de analizar o predecir aspectos relativos a mi salud.

b. Que los tratamientos indicados se encuentran legitimados en el consentimiento otorgado por mi parte.

c. Que mis datos personales, obtenidos mediante la cumplimentación de este formulario, así como los resultantes de mi participación en el proyecto serán conservados durante el tiempo necesario para el desarrollo de esta investigación, que se estima de seis meses, siendo posteriormente destruidos, sin que puedan ser conservados sin haber sido previamente anonimizados. En cualquier caso, no podrán ser cedidos sin mi consentimiento expreso y no lo otorgo en este acto.

d. Que puedo contactar con el Delegado de Protección de Datos de FUSP-CEU, dirigiendo mi petición por escrito a la dirección postal C/ Tutor nº 35 - 28008 Madrid o a la dirección de correo electrónico dpd@ceu.es.

e. Que de acuerdo con los derechos que me confiere la normativa vigente en protección de datos podré dirigirme a la Autoridad de Control competente para presentar la reclamación que considere oportuna, así como también podré ejercer los derechos de acceso, rectificación, limitación de tratamiento, supresión, portabilidad y oposición al tratamiento de mis datos de carácter personal y retirar el consentimiento prestado para el tratamiento de los mismos, dirigiendo mi petición al investigador responsable en la dirección de contacto que figura en este documento.

7. Estoy de acuerdo en que mi consentimiento por escrito y otros datos estén a disposición del proyecto de investigación clínico en el que estoy participando, y del investigador responsable del mismo, Noemí Valtueña Gimeno, pero siempre respetando la confidencialidad y la garantía de que mis datos no estarán disponibles públicamente de forma que pueda ser identificado.

8. Los datos recogidos para este estudio serán incluidos, con los de otras personas que participen en este estudio, en una base de datos de carácter personal de la Universidad CEU Cardenal Herrera a la que sólo los investigadores aprobados para este proyecto tendrán acceso, estando todos ellos sometidos al secreto inherente a su profesión o derivado de un acuerdo de confidencialidad.

9. Firmo este documento de información y consentimiento de forma voluntaria para manifestar mi deseo de participar en este estudio de investigación sobre los efectos de la terapia manual diafragmática en el equilibrio dinámico de bailarines profesionales, hasta que decida lo contrario. Al firmar este consentimiento no renuncio a ninguno de mis derechos. Recibiré una copia de este documento para guardarlo y poder consultarlo en el futuro.

Por tanto, doy mi conformidad y consentimiento para a que realice el estudio detallado con la ayuda del personal que sea necesario con la debida cualificación y especialización.

El participante

(Firma) Nombre, Apellidos

Valencia, a ……… de ………………… de

**AUTORIZACIÓN DEL FAMILIAR O TUTOR**

Ante la imposibilidad de D./Dña.

con DNI de prestar autorización para los tratamientos explicitados en el presente documento de forma libre, voluntaria, y consciente.

D./Dña.

con DNI

En calidad de (marido, esposa, hijo, hermano, tutor legal, familiar, allegado, cuidador), decido, dentro de las opciones clínicas disponibles, dar mi conformidad libre, voluntaria y consciente a la técnica descrita para los tratamientos explicitados en el presente documento.

_________, _____de _______________________de____________

**INVESTIGADOR**

D./Dña.

con DNI

Correo electrónico:

Teléfono:

Investigador de la Universidad CEU-Cardenal Herrera de Valencia, declaro haber facilitado al participante del estudio y/o persona autorizada, toda la información necesaria para la realización de la intervención explicitada en el presente documento y declaro haber confirmado, inmediatamente antes de la aplicación de la técnica, que el participante no incurre en ninguno de los casos de contraindicación relacionados anteriormente, así como haber tomado todas las precauciones necesarias para que la intervención correcta.

___________, _____de _______________________de____________

**REVOCACIÓN DEL CONSENTIMIENTO INFORMADO**

**D/Dña.**

con DNI

Revoco el consentimiento prestado en fecha de de

Y no deseo proseguir el tratamiento que doy en esta fecha por finalizado.

______________, _____de _______________________de____________

**ANEXO II. FASE DE ENTRENAMIENTO DE FUERZA**

**Entrenamiento de fuerza clásico**

| NIVEL | EJERCICIO | DOSIFICACIÓN |
| --- | --- | --- |
| NIVEL 01 | - F/Ext cadera en DS Theraband: 15rep cada pierna - Ext cadera en BipeTheraband: 12rep cada pierna - Bíceps en BipeTheraband: 15rep cada pierna - Tríceps en BipeTheraband: 15rep cada pierna | *20’ de Ejercicios*  *TABATA de 3 series*  *20’’ rest entre ejercicios*  *60’’ rest entre series* |
| NIVEL 02 | - Squats en Bipe: 15rep - Ext cadera en DP con lastre: 12rep cada pierna - Bíceps en BipeTheraband (+): 15rep cada pierna - Tríceps en BipeTheraband (+): 15rep cada pierna | *20’ de Ejercicios*  *TABATA de 3 series*  *20’’ rest entre ejercicios*  *60’’ rest entre series* |
| NIVEL 03 | - Lunge estático en Bipe: 12rep cada pierna - Monster-walk por aula Theraband: 30’’ cada pierna - Abdominales Sahrmann (nivel 1-2) - Plank en DP: 30’’ | *20’ de Ejercicios*  *TABATA de 3 series*  *20’’ rest entre ejercicios*  *60’’ rest entre series* |
| NIVEL 04 | - Lunge en pasillo con pesa: 12rep cada pierna - Monster-walk por aula Theraband (+): 30’’ cada pierna - Abdominales Sahrmann (nivel 2-3) - Plank en DP con elevación de pierna: 30’’ | *20’ de Ejercicios*  *TABATA de 3 series*  *20’’ rest entre ejercicios*  *60’’ rest entre series* |

**Entrenamiento de fuerza neuromuscular**

| NIVEL | EJERCICIO | DOSIFICACIÓN |
| --- | --- | --- |
| NIVEL 01 | - Cuarto de sentadilla asistido: 15 rep - Puente de glúteo no modificado: 12 rep - Flexión de MM SS asistida bilateral: 15 rep - Bipedestación sobre BOSU: 30 “ 2 rep | *20’ de Ejercicios*  *TABATA de 3 series*  *20’’ rest entre ejercicios*  *60’’ rest entre series* |
| NIVEL 02 | - Cuarto de sentadilla sin asistencia: 15 rep - Puente de glúteo modificado: 12 rep - Flexión MMSS Unilateral: 12 rep - Bipedestación sobre air cushion: 30 “ 2 rep | *20’ de Ejercicios*  *TABATA de 3 series*  *20’’ rest entre ejercicios*  *60’’ rest entre series* |
| NIVEL 03 | - Lunge sobre BOSU: 12 rep con cada pierna - Pivotaje contra resistencia (glúteo medio): 12 rep con cada pierna - Trabajo abdominal con disociación de MMSS: 25 rep - Step subida lateral: 12 rep con cada pierna | *20’ de Ejercicios*  *TABATA de 3 series*  *20’’ rest entre ejercicios*  *60’’ rest entre series* |
| NIVEL 04 | - Flexo extensión de cadera en todo el rango con carga : 12 rep cada pierna - Starsexcursion test: 30” cada pierna - Control abdominal en balón de Bobath: 30” 2 rep - Subida de escalones modificada con carga: 15 rep | *20’ de Ejercicios*  *TABATA de 3 series*  *20’’ rest entre ejercicios*  *60’’ rest entre series* |

|  |  |  |
| --- | --- | --- |
|  |  |  |
|  |  |  |
|  |  |  |
