# Supplementary material for: Effects of neuromuscular training compared to classic strength-resistance training in patients with acute coronary syndrome: A study protocol for a randomized controlled trial
Source: PLoS One. 2020 Dec 23;15(12):e0243917. doi: 10.1371/journal.pone.0243917 (PMC7757882; doi:10.1371/journal.pone.0243917)
Supplement: S2 Protocol — (DOCX) [file pone.0243917.s004.docx]

**EFFECT OF NEUROMUSCULAR TRAINING ON FUNCTIONAL CAPACITIES IN PATIENTS WITH ACUTE CORONARY SYNDROME**

INTRODUCTION

Acute myocardial infarction (AMI) is defined as a clinical picture produced by ischemia and subsequent death of a more or less extensive portion of the myocardium, as a consequence of the obstruction of a coronary artery. When flow obstruction occurs, the blood supply to the myocardial tissue is suppressed, producing an oxygen deficit that, if maintained over time, produces non-reversible necrosis of the affected myocardial area^1,2^.

In the past decade, ischemic heart disease caused up to 29% of deaths in industrialized countries, becoming the main cause of death, a figure that will remain in 2020 according to the latest projections^3^. However, a decrease in mortality from ischemic heart disease has been observed, related to a lower incidence and to the improvements introduced in recent years both in the acute phase treatment of acute myocardial infarction and in primary prevention ^4,5^.

The clinical manifestations of heart disease are extensive, with chest pain as the main sign, presenting with strong intensity and without yielding to rest. The radiation of this pain can be variable (interscapular, mandibular, cervical, upper limbs, etc.) and can be accompanied by nonspecific signs such as dyspnea, sweating and dizziness, among others^1,6^.

In addition to the acute symptoms of the disease, after AMI patients have to learn to live with the consequences of the disease and its treatment, which can lead to a deterioration in health-related quality of life (HRQoL) ^7, 8^. There is no consensus on the concept of HRQL, but what the different authors agree on is that it is a complex and multidimensional concept that acquires great importance when evaluating the impact of a disease or health problem, in which case we are talking about HRQL. This concept encompasses aspects such as physical symptoms; cognitive function and psychological well-being; performance or role and social welfare; the global state of health; the perception of care and personal constructs^8,9^.

Sexual activity is an aspect that affects HRQoL in patients with acute myocardial infarction (AMI) and their spouses^10,11^. Both the usual treatment of patients, as well as cardiovascular adaptations during sexual intercourse, can aggravate the symptoms of heart disease by increasing oxygen consumption^12-14^. This energy consumption during sexual intercourse in the usual position and with the usual partner is equivalent to 3-4 METs (Metabolic Equivalent or Metabolic Equivalent of Task), a MET being the oxygen consumption per minute of a person at rest in relation to their body weight. Although sexual activity is a risk factor for patients who have suffered AMI, patients who can exercise whose energy consumption is equivalent to or greater than 5 METS, are in a position to resume sexual activity^12,15^.

The importance of a heart-healthy lifestyle, which includes physical exercise, has been described and evidenced in both adult and pediatric populations^16^. The implementation of comprehensive evaluation programs of cardiopulmonary function and the response to physical activity, as well as the design of cardiac rehabilitation based on this evaluation, can favor the quality of life of patients with heart disease after acute myocardial infarction, in addition to be very useful for evaluating the clinical status and prognosis of the patient.

Cardiac rehabilitation programs (CRP) are multifactorial action systems recommended by the World Health Organization since the 1960s. There is scientific evidence that these programs not only favor the control of cardiovascular risk factors (CVRF), but also act on quality of life, favoring work reintegration and reducing the morbidity and mortality of patients, in addition to demonstrating in a way its viability has been unequivocal as its effectiveness has been verified at the cost-benefit level^17-19^.

Contraindications to performing CRPs have been reduced in recent years thanks to a better understanding of the results and dangers inherent in exercising. Those that could be considered as absolute are reduced to dissecting aortic aneurysms and severe obstruction of the left ventricular outflow tract without surgical indication^1,4^.

The CRPs are divided into three phases. Phase I includes the hospital stay, which can also occur with a patient in the phase before and after cardiovascular surgery. This phase includes early mobilization and respiratory exercises, as well as control of CVRF and psychological action. Phase II lasts approximately two months and begins from the moment of hospital discharge. The clinical data and results of the examinations (such as echocardiography, ergometry, nuclear medicine, etc.) allow patients to be classified at different risk levels. This phase consists of 20-24 sessions with two or three face-to-face sessions in the rehab center, including a 10-minute warm-up period, a 30-40-minute aerobic workout, and a 10-minute recovery phase. The intensity of the exercise is determined according to the data of a stress test, and is increased as a function of the chronotropic and tension response of the patient to the training, in addition to her subjective perception of effort. Phase III is maintained for the rest of the patient's life. It is performed out-of-hospital and, after a written report from the cardiologist, the cardiovascular patient continues with the established physical exercise guidelines, control of CVRF, and socio-occupational and psychological aspects in centers equipped with the material and human equipment necessary for the continuation of his rehabilitation^1,4,16,18,20^.

In recent years, the study of movement dysfunction and its influence on the efficiency of sports gesture performance, especially of the lower limb, has undergone considerable development. From the studies by Sahrmann et al.^21-25^, to the most recent ones carried out by Comerford et al.^26,27^, the importance of neuromuscular control to improve movement quality and prevent injuries derived from this lack of control has been evidenced. This type of training focuses on several key elements such as core stability, performing them in a functional posture, lower limb strength work, and functional exercises^28^.

Despite the fact that the bulk of the evidence on the benefit of this type of work is in the world of sport due to its importance in preventing injuries and improving control and proprioception of the joint segments, other studies are testing the effectiveness of this work for other populations^29,30^. For example, the GLA: DTM program, a neuromuscular training program run by physiotherapists throughout Denmark is being carried out to improve the symptoms caused by hip osteoarthritis in adult and elderly populations. However, its authors point out the need to study the potential of this type of work not only in pathologies of neuromuscular etiology, but also in other chronic or supervening diseases and in the ability to improve the general condition of the patient^31^. For this reason, we propose this study to know the possible benefits of this work compared to a traditional force protocol.

OBJECTIVES

The general objective of the study is based on verifying whether neuromuscular training is more effective compared to the classic strength training of Cardiac Rehabilitation Programs to improve the functional capacities in patients after acute myocardial infarction.

The specific objectives of this project are:

- Analyze the effect of the intervention on functional parameters of lower limbs

- Determine the effect of the program on variables of strength of respiratory and peripheral muscles

- Analyze the impact of a cardiac rehabilitation program on the quality of life of patients who have suffered an AMI

- Determine the variables that influence the sexual activity of patients after AMI and the impact on their quality of life

METHODS

**Design**

The study is presented as a randomized controlled trial, an experimental scientific procedure characterized by the random distribution of the members of a suitable population group into an intervention group and a control group, comparing a specific impact between the groups after a period established in advance.

Its design is a pre-post, based on the measurement and comparison of the variables to be studied before and after the exposure of the participants to the experimental intervention.

**Participants**

Participants will be selected based on the following criteria:

Inclusion criteria:

• Subjects diagnosed with acute coronary syndrome with or without ST segment elevation (NSTEACS / NSTEACS), with a Low or Medium risk stratification according to the stress tests performed by the cardiology service of the IMED Valencia Hospital.

• Medical prescription.

• Consent signed by the patient (Annex I).

Exclusion criteria:

• Pathologies that contraindicate exercise: acute inflammatory cardiac disease, severe or moderate LV or RV dysfunction, decompensated HF, serious arrhythmias documented in ICD, significant coronary artery disease, HTN> 40mmHg or requiring treatment with vasodilators, gradient aortic stenosis resting systolic> 50mmHg, pulmonary stenosis with resting systolic gradient <50mmHg, systemic hypertension> 95% percentile, baseline O2 Sat <90%, severe AV valve failure, severe acute kidney disease, acute hepatitis and active infection.

• Alterations in ergospirometry: severe exercise-induced arrhythmias, ST depression with effort, exaggerated hypertensive response, hypotension on exertion and chest pain.

**Intervention: training program**

Phase II of the cardiac rehabilitation program will be based on the F.I.T.T.^32,33^ model, where the main parameters are the frequency of training, the intensity of the exercise, the duration of the sessions / complete program, and the type of exercise proposed:

- FREQUENCY. 2 sessions per week in a cardiac rehabilitation gym. In addition, the work will be complemented with a pattern of home exercise.

- INTENSITY. Intensity based on maximum oxygen consumption (VO2) and maximum heart rate (HR), calculated based on the ergospirometric tests performed. The Training HR (FCE) will be that given by the ventilatory thresholds obtained in the Wasserman curves.

- TIME. The sessions will last approximately 60 minutes (approximately 40 minutes of them correspond to the training phase). The program will include a total of 20 sessions (2 sessions a week for approximately 10 weeks).

- TYPE. The type of work indicated in cardiac rehabilitation programs in the aerobic phase will be the continuous and interval modality, which will be established depending on the risk stratification of the patient.

All patients will be monitored through a heart rate and O2 saturation recognition device. In addition, the patient's subjective perception of effort will be monitored at all times using the modified Borg Scale, both at the level of dyspnea and lower limb fatigue.

The sessions will be structured as follows:

Warm-up Phase (10 minutes):

- Respiratory exercises: Diaphragm + respiratory muscles training

- Low-intensity walking

Training Phase (40 minutes):

- Aerobic Exercise Phase on treadmill / cycle ergometer (20 minutes)

- Strength Exercise Phase (20 minutes) (Annex II)

Cooldown Phase (10 minutes):

- Low-intensity walking

- Stretching

**Outcome measures:**

Sociodemographic variables: age, sex, educational level, type of work, marital status, time spent with the partner.

Dependent and independent variables:

The evaluation of these variables will be carried out in conjunction with the cardiology service of the IMED Valencia Hospital. At the Hospital IMED, tests related to the patient's risk stratification (ergospirometry, echocardiography and biochemical profile) will be performed, and at CEU Cardenal Herrera University the patient's respiratory and functional capacities will be evaluated (pressure measurement, incremental Shuttle test, evaluation of strength, functional parameters of the lower limb), in addition to quality of life and sexual dysfunction questionnaires.

All variables will be evaluated at 3 different times: pre-intervention, immediate post-intervention and one year follow-up.

- Cardiology Service (Hospital IMED Valencia):

- Cardiopulmonary exercise testing (CPET), evaluated by ergospirometry. The stress test with oxygen consumption consists of subjecting the patient to physical exercise with a controlled and progressive workload, taking measurements before, during, and after carrying out said work, and subsequently analyzing the data obtained . This test provides us with information about their cardiac, pulmonary, musculoskeletal function, and the response of all of them to physical exercise, constituting essential information for a correct evaluation of the patient's situation, establishment of a prognosis, and a good point of evaluation. Departure for the design of cardiac rehabilitation programs in the population after acute myocardial infarction. Among the necessary equipment, a cycle ergometer / treadmill is essential, as well as measurement and analysis equipment, such as a cardiopulmonary exercise tower, metabolic cart, electrocardiogram, pulse oximeter, blood pressure monitor, and additional equipment (resuscitation cart, stretcher of examination, oxygen bullet) and accessories^34^.
- Ejection Fraction, measured by echocardiography. Ejection fraction is defined as the stroke volume divided by the ventricular volume at the end of diastole. In the left ventricle, it usually has a value of about two-thirds (55 to 65%). In patients who have suffered an acute myocardial infarction, the ejection fraction serves as an indicator of risk, along with other elements. Thus, the patient's risk can be stratified into Low, Medium and High.
- Biochemistry. Cardiac markers are used during the first hours for the diagnosis and evaluation of myocardial damage secondary to an AMI. In addition to the evaluation of these markers, numerous parameters can be altered in a patient with cardiovascular risk factors, such as in those with a history of dyslipidaemia or diabetes. Standard follow-up of patients includes biochemical analysis of triglyceride levels, total cholesterol, HDL and LDL cholesterol, and glycosylated hemoglobin. This study will also include inflammation markers such as C-reactive protein and interleukin 6, and NT-proBNP in those patients with associated heart failure. Likewise, iron metabolism parameters (serum iron, ferritin, transferrin saturation index, serum transferrin) will be included.

- Rehabilitation Service (CEU Cardenal Herrera University):

- Incremental Shuttle Walking Test (ISWT). It is a test derived from the "20-meter Shuttle run test" adapted to cardiac patients, such as those with coronary heart disease. The ISWT has demonstrated its effectiveness in the field of cardiac rehabilitation, in addition to being correlated with the maximum VO2 and metabolic equivalents of the cardiovascular patient. The patient must walk between two marks 9 meters apart, progressively increasing their speed^35-37^.

Functional capacities:

- 30 ’’ stand to sit test. It measures the maximum number of squats the patient is capable of performing in 30 seconds. The objective is to assess muscular endurance and rapid changes between concentric and eccentric contraction of the lower limb muscles. From a sitting position, the patient fully sits up in a standing position and returns to a full sitting position as many times as possible in the established time^38^.
- Monopodal quarter squat. The subject is asked to flex the knee in single leg stance. The evaluator scores performance as defined by Comerford and Motram^26,39^.
- Strength of hip extensors. It will be done using a hand-held dynamometer, carrying out 3 measurements of a concentric isometric contraction^38,40^.
- Chester Step Test. In this submaximal test, the patient must go up and down a step to the rhythm of a metronome, progressively increasing the speed. The height of the step will depend on the age and previous physical activity of the subject. This test has proven to be a good indicator of cardiovascular risk factors and a predictor of cardiovascular risk^41,42^.
- Maximum static inspiratory pressure (PIM, cmH2O) / Maximum static expiratory pressure (PEM, cmH2O), or maximum positive and negative pressure, respectively, that can generate and maintain the entire respiratory musculature, against an occluded circuit, for at least one 1 second^43^. It is determined at or near total lung capacity and residual volume^44^. A minimum of 3 measurements are made, 1 minute apart, and the highest value of the 3 extracted is selected for the results. The MicroRPM device (Carefusion, U.K.) will be used.

- Nursing Service (CEU Cardenal Herrera University):

- EuroQol (EQ-5D). It consists of two parts. The first corresponds to the description of the state of health in five dimensions (mobility, personal care, daily activities, pain / discomfort and anxiety / depression). In the second part of the questionnaire, the subject must score on a millimeter visual analog scale of their health status at the time they self-classify or self-perceive their personal status, having at the ends of the scale the worst health status (0) and the better health (100)^45^.
- The self-administered Sexual Health Inventory for Men questionnaire, validated and consisting of 5 items, will also be used in this study. This questionnaire is an abbreviated version of the International Index of Erectile Function Test and assesses erectile dysfunction in men^46^.

STATISTICAL ANALYSIS

The data analysis will be carried out with the statistical program IBM SPSS for Windows, version 24.0. Armonk, NY: IBM Corp.

The sample size calculation will be carried out based on the results of a previous pilot study.

The comparison of the results obtained between the Intervention group and the Control group before and after the intervention will be carried out using a mixed factorial model (Split-plot). The intra-subject factor will be time (PRE, POST, POST1). The inter-subject factor will be the group, with two levels: Intervention and Control.

A 95% confidence interval will be considered for the difference and statistical significance will be established at p <0.05.

REFERENCES

(1) Velasco JA, Cosín J, Maroto JM, Muñiz J, Casasnovas JA, Plaza I, et al. Guías de práctica clínica de la Sociedad Española de Cardiología en prevención cardiovascular y rehabilitación cardíaca. Revista Española de Cardiología 2000;53(8):1095-1120.

(2) Maroto, JM. De Pablo, C. Artigao, R. Rehabilitación Cardíaca. Sociedad Española de Cardiología. Olalla Cardiología Ediciones 2009:500-549.

(3) Maroto Montero JM, Artigao Ramírez R, Morales Durán MD, de Pablo Zarzosa C, Abraira V. Rehabilitación cardíaca en pacientes con infarto de miocardio. Resultados tras 10 años de seguimiento. Revista española de cardiología 2005;58(10):1181-1187.

(4) de la Cuerda, Roberto Cano, Diego IMA, Martín JJA, Sánchez AM, Page JCM. Programas de rehabilitación cardiaca y calidad de vida relacionada con la salud. Situación actual. Revista Española de Cardiología 2012;65(1):72-79.

(5) Wood DA. Clinical reality of coronary prevention guidelines: a comparison of EUROASPIRE I and II in nine countries. The Lancet 2001;357(9261):995-1001.

(6) Kassab Y, Hassan Y, Aziz NA, Ismail O, AbdulRazzaq H. Patients’ adherence to secondary prevention pharmacotherapy after acute coronary syndromes. International journal of clinical pharmacy 2013;35(2):275-280.

(7) Medrano MJ, Cerrato E, Boix R, Delgado-Rodríguez M. Factores de riesgo cardiovascular en la población española: metaanálisis de estudios transversales. Medicina clínica 2005;124(16):606-612.

(8) Grau M, Elosua R, de Leon AC, Guembe MJ, Baena-Díez JM, Alonso TV, et al. Factores de riesgo cardiovascular en España en la primera década del siglo XXI: análisis agrupado con datos individuales de 11 estudios de base poblacional, estudio DARIOS. Revista Española de Cardiología 2011;64(4):295-304.

(9) Bauer LK, Caro MA, Beach SR, Mastromauro CA, Lenihan E, Januzzi JL, et al. Effects of depression and anxiety improvement on adherence to medication and health behaviors in recently hospitalized cardiac patients. Am J Cardiol 2012;109(9):1266-1271.

(10) Thylén I, Brännström M. Intimate relationships and sexual function in partnered patients in the year before and one year after a myocardial infarction: a longitudinal study. European Journal of Cardiovascular Nursing 2015;14(6):468-477.

(11) Rosman L, Cahill JM, McCammon SL, Sears SF. Sexual health concerns in patients with cardiovascular disease. Circulation 2014 Feb 4;129(5):e313-6.

(12) Levine GN, Steinke EE, Bakaeen FG, Bozkurt B, Cheitlin MD, Conti JB, et al. Sexual activity and cardiovascular disease: a scientific statement from the American Heart Association. Circulation 2012 Feb 28;125(8):1058-1072.

(13) Lim S, Sim Ds, Han J. The factors associated with sexual recovery in male patients with acute myocardial infarction under phase II cardiac rehabilitation. J Clin Nurs 2016;25(19-20):2827-2834.

(14) Bispo GS, de Lima Lopes J, de Barros AL. Cardiovascular changes resulting from sexual activity and sexual dysfunction after myocardial infarction: integrative review. J Clin Nurs 2013;22(23-24):3522-3531.

(15) Dahabreh IJ, Paulus JK. Association of episodic physical and sexual activity with triggering of acute cardiac events: systematic review and meta-analysis. JAMA 2011;305(12):1225-1233.

(16) Espinosa J, De Teresa C, Navas J. Rehabilitación en afecciones cardíacas. Afecciones médicas en fisioterapia. 1ª ed. Málaga: Spicum; 1999. p. 467-477.

(17) Amigo Castañeda P, Amigo González R, Rodríguez Díaz M, Castañeda Gueimonde CM. Modificación de algunos factores de riesgo coronario después de la rehabilitación física. Revista Médica Electrónica 2010;32(3):0-0.

(18) Caliani JSE, Navas JCB. La rehabilitación cardiaca en el centro de salud. Intervención del fisioterapeuta. Rehabilitación cardíaca y atención primaria. 2ª ed. Madrid: Ed. Médica Panamericana; 2002. p. 95-112.

(19) Plaza Pérez I. Estado actual de los programas de prevención secundaria y rehabilitación cardiaca en España. Revista Española de Cardiología 2003;56(08):757-760.

(20) O’Donnell CJ, Elosua R. Factores de riesgo cardiovascular. Perspectivas derivadas del Framingham Heart Study. Revista española de Cardiología 2008;61(3):299-310.

(21) Van Dillen LR, Sahrmann SA, Norton BJ, Caldwell CA, Fleming D, McDonnell MK, et al. Effect of active limb movements on symptoms in patients with low back pain. Journal of Orthopaedic & Sports Physical Therapy 2001;31(8):402-418.

(22) Van Dillen LR, McDonnell MK, Fleming DA, Sahrmann SA. Effect of knee and hip position on hip extension range of motion in individuals with and without low back pain. Journal of Orthopaedic & Sports Physical Therapy 2000;30(6):307-316.

(23) Van Dillen LR, Maluf KS, Sahrmann SA. Further examination of modifying patient-preferred movement and alignment strategies in patients with low back pain during symptomatic tests. Man Ther 2009;14(1):52-60.

(24) Van Dillen LR, Sahrmann SA, Norton BJ, Caldwell CA, McDonnell MK, Bloom NJ. Movement system impairment-based categories for low back pain: stage 1 validation. Journal of Orthopaedic & Sports Physical Therapy 2003;33(3):126-142.

(25) Van Dillen LR, Sahrmann SA, Norton BJ, Caldwell CA, Fleming DA, McDonnell MK, et al. Reliability of physical examination items used for classification of patients with low back pain. Phys Ther 1998 Sep;78(9):979-988.

(26) Comerford M, Mottram S. Kinetic control: the management of uncontrolled movement. : Elsevier Australia; 2012.

(27) Comerford MJ, Mottram SL. Movement and stability dysfunction–contemporary developments. Man Ther 2001;6(1):15-26.

(28) Ageberg E, Link A, Roos EM. Feasibility of neuromuscular training in patients with severe hip or knee OA: the individualized goal-based NEMEX-TJR training program. BMC musculoskeletal disorders 2010;11(1):126.

(29) Ageberg E, Nilsdotter A, Kosek E, Roos EM. Effects of neuromuscular training (NEMEX-TJR) on patient-reported outcomes and physical function in severe primary hip or knee osteoarthritis: a controlled before-and-after study. BMC musculoskeletal disorders 2013;14(1):232.

(30) Steib S, Rahlf AL, Pfeifer K, Zech A. Dose-Response Relationship of Neuromuscular Training for Injury Prevention in Youth Athletes: A Meta-Analysis. Frontiers in physiology 2017;8:920.

(31) Skou ST, Roos EM. Good Life with osteoArthritis in Denmark (GLA: D™): evidence-based education and supervised neuromuscular exercise delivered by certified physiotherapists nationwide. BMC musculoskeletal disorders 2017;18(1):72.

(32) American College of Sports Medicine. ACSM's guidelines for exercise testing and prescription. : Lippincott Williams & Wilkins; 2013.

(33) Billinger SA, Boyne P, Coughenour E, Dunning K, Mattlage A. Does aerobic exercise and the FITT principle fit into stroke recovery? Current neurology and neuroscience reports 2015;15(2):519.

(34) Wasserman K, Hansen JE, Sue DY, Stringer WW, Whipp BJ. Principles of exercise testing and interpretation: including pathophysiology and clinical applications. Medicine & Science in Sports & Exercise 2005;37(7):1249.

(35) Casillas J, Hannequin A, Besson D, Bénaïm S, Krawcow C, Laurent Y, et al. Walking tests during the exercise training: specific use for the cardiac rehabilitation. Annals of physical and rehabilitation medicine 2013;56(7-8):561-575.

(36) Hanson LC, Taylor NF, McBurney H. The 10 m incremental shuttle walk test is a highly reliable field exercise test for patients referred to cardiac rehabilitation: a retest reliability study. Physiotherapy 2016;102(3):243-248.

(37) Pichurko BM. Exercising your patient: which test(s) and when? Respir Care 2012 Jan;57(1):100-10; discussion 110-3.

(38) Dobson F, Hinman R, Roos EM, Abbott J, Stratford P, Davis A, et al. OARSI recommended performance-based tests to assess physical function in people diagnosed with hip or knee osteoarthritis. Osteoarthritis and cartilage 2013;21(8):1042-1052.

(39) Weeks BK, Carty CP, Horan SA. Kinematic predictors of single-leg squat performance: a comparison of experienced physiotherapists and student physiotherapists. BMC musculoskeletal disorders 2012;13(1):207.

(40) Andrews AW, Thomas MW, Bohannon RW. Normative values for isometric muscle force measurements obtained with hand-held dynamometers. Phys Ther 1996;76(3):248-259.

(41) Buckley JP, Sim J, Eston RG, Hession R, Fox R. Reliability and validity of measures taken during the Chester step test to predict aerobic power and to prescribe aerobic exercise. Br J Sports Med 2004 Apr;38(2):197-205.

(42) Gray BJ, Stephens JW, Williams SP, Davies CA, Turner D, Bracken RM, et al. Cardiorespiratory fitness is a stronger indicator of cardiometabolic risk factors and risk prediction than self-reported physical activity levels. Diabetes and Vascular Disease Research 2015;12(6):428-435.

(43) Black LF, Hyatt RE. Maximal respiratory pressures: normal values and relationship to age and sex. Am Rev Respir Dis 1969;99(5):696-702.

(44) American Thoracic Society/European Respiratory Society. ATS/ERS Statement on respiratory muscle testing. Am J Respir Crit Care Med 2002 Aug 15;166(4):518-624.

(45) Herdman M, Badía X, Berra S. El EuroQol-5D: una alternativa sencilla para la medición de la calidad de vida relacionada con la salud en atención primaria. Atención primaria 2001;28(6):425-429.

(46) Cappelleri JC, Siegel RL, Glasser DB, Osterloh IH, Rosen RC. Relationship between patient self-assessment of erectile dysfunction and the sexual health inventory for men. Clin Ther. 2001;23(10):1707–19.

ANNEX I DOCUMENT OF CONSENT FOR PARTICIPATION IN A RESEARCH PROJECT

Effect of neuromuscular training on functional capacities in patients after acute myocardial infarction / MI of the project: Noemí Valtueña Gimeno

Mr / Mrs _______________________________________________________

With ID No. ____________________

Freely and voluntarily

MANIFEST:

1. I have read and understood the information sheet under study.

2. I have had the opportunity to ask questions.

3. My questions have been answered to my satisfaction.

4. I have received sufficient information about the study and the tests to be carried out.

5. I understand that participation is voluntary and I can leave the study at any time without having to give an explanation and without affecting my medical care.

6. In accordance with the provisions of Regulation (EU) 2016/679 of the European Parliament and of the Council, of April 27, 2016, regarding the protection of natural persons with regard to the processing of personal data and freedom of circulation of these data and by which Directive 95/46 / CE is repealed, I have been informed that my personal data, obtained by completing this form as well as those resulting from my participation in the project, will be treated under the responsibility of the SAN PABLO CEU UNIVERSITY FOUNDATION (hereinafter, FUSP-CEU), in order to manage my participation in this research project. In addition, I have been informed of the following aspects:

a. That profiling is planned in order to analyze or predict aspects related to my health.

b. That the indicated treatments are legitimized in the consent granted by me.

c. That my personal data, obtained by completing this form, as well as those resulting from my participation in the project will be kept for the time necessary for the development of this research, which is estimated to be six months, being subsequently destroyed, without being able to be preserved without having been previously anonymized. In any case, they cannot be transferred without my express consent and I do not grant it in this act.

d. That I can contact the Data Protection Delegate of FUSP-CEU, directing my request in writing to the postal address C / Tutor nº 35 - 28008 Madrid or to the email address dpd@ceu.es.

e. That in accordance with the rights conferred on me by current data protection regulations, I may contact the competent Control Authority to present the claim that I consider appropriate, as well as I may exercise the rights of access, rectification, limitation of treatment, deletion, portability and opposition to the treatment of my personal data and withdraw the consent given for the treatment thereof, directing my request to the responsible researcher at the contact address that appears in this document.

7. I agree that my written consent and other data are available to the clinical research project in which I am participating, and to the researcher responsible for it, Noemí Valtueña Gimeno, but always respecting confidentiality and the guarantee that my data will not be publicly available so that you can be identified.

8. The data collected for this study will be included, together with those of other people participating in this study, in a personal database of the CEU Cardenal Herrera University to which only researchers approved for this project will have access, being all of them subject to the secrecy inherent to their profession or derived from a confidentiality agreement.

9. I sign this information and consent document voluntarily to express my desire to participate in this research study on the effects of manual diaphragmatic therapy on the dynamic balance of professional dancers, until I decide otherwise. By signing this consent I do not waive any of my rights. I will receive a copy of this document to keep for future reference.

Therefore, I give my consent and consent for you to carry out the detailed study with the help of the necessary personnel with the due qualifications and specialization.

The participant

(Signature) Name, Surname

Valencia, on ……… of ………………… of

AUTHORIZATION OF THE FAMILY OR GUARDIAN

Given the impossibility of Mr./Mrs.

with ID No. to provide authorization for the treatments specified in this document freely, voluntarily, and consciously.

Mr./Mrs.

with ID No.

As (husband, wife, son, brother, legal guardian, relative, close friend, caregiver), I decide, within the clinical options available, to give my free, voluntary and conscious consent to the technique described for the treatments specified in this present document.

_________, _____of _______________________of____________

INVESTIGATOR

Mr./Mrs.

with ID No.

Email:

Telephone:

Researcher at the CEU-Cardenal Herrera University of Valencia, I declare that I have provided the study participant and / or authorized person with all the information necessary to carry out the intervention specified in this document and I declare that I have confirmed, immediately before the application of the technique, that the participant does not incur any of the contraindication cases listed above, as well as having taken all the necessary precautions for the correct intervention.

___________, _____of _______________________of____________

REVOCATION OF INFORMED CONSENT

Mr. / Ms.

with ID No.

I revoke the consent given on the date of

And I do not want to continue the treatment that I give on this date as finished.

______________, _____of _______________________of____________

ANNEX II. STRENGTH TRAINING PHASE

Classic strength training

| LEVEL | EXERCISE | DOSAGE |
| --- | --- | --- |
| LEVEL 01 | - Hip F/Ext in supine Theraband: 15rep each leg - Hip Ext in Bipe Theraband: 12rep each leg - Bíceps in BipeTheraband: 15rep each leg - Tríceps in BipeTheraband: 15rep each leg | *20’ Exercises*  *TABATA 3 series*  *20’’ rest between exercises*  *60’’ rest between exercises* |
| LEVEL 02 | - Squats in Bipe: 15rep - Weighted Hip Ext in prone: 12rep each leg - Bíceps en BipeTheraband (+): 15rep each leg - Tríceps en BipeTheraband (+): 15rep each leg | *20’ Exercises*  *TABATA 3 series*  *20’’ rest between exercises*  *60’’ rest between exercises* |
| LEVEL 03 | - Static lunge in Bipe: 12rep each leg - Monster-walk Theraband: 30’’ each leg - Abdominales Sahrmann (nivel 1-2) - Plank in prone: 30’’ | *20’ Exercises*  *TABATA 3 series*  *20’’ rest between exercises*  *60’’ rest between exercises* |
| LEVEL 04 | - Weighted Lunge: 12rep each leg - Monster-walk Theraband (+): 30’’ each leg - Abdominales Sahrmann (nivel 2-3) - Plank in prone raising leg: 30’’ | *20’ Exercises*  *TABATA 3 series*  *20’’ rest between exercises*  *60’’ rest between exercises* |

Neuromuscular strength training

| LEVEL | EXERCISE | DOSAGE |
| --- | --- | --- |
| LEVEL 01 | - Assisted quarter squat: 15 rep - Unmodified gluteal bridge: 12 rep - Bilateral assisted upper limb flexión: 15 rep - Standing on BOSU: 30 “ 2 rep | *20’ Exercises*  *TABATA 3 series*  *20’’ rest between exercises*  *60’’ rest between exercises* |
| LEVEL 02 | - Non-assisted quarter squat: 15 rep - Modified gluteal bridge:12 rep - Unilateral upper limb flexión: 12 rep - Standing on air cushion: 30 “ 2 rep | *20’ Exercises*  *TABATA 3 series*  *20’’ rest between exercises*  *60’’ rest between exercises* |
| LEVEL 03 | - Lunge on BOSU: 12 rep each leg - Pivot agains resistance (gluteus medius): 12 rep each leg - Abdominal with upper limb disociation: 25 rep - Lateral Step: 12 rep each leg | *20’ Exercises*  *TABATA 3 series*  *20’’ rest between exercises*  *60’’ rest between exercises* |
| LEVEL 04 | - Weighted Hip Flexo- extensión: 12 rep each leg - Starsexcursion test: 30” each leg - Abdominal control on Bobath ball: 30” 2 rep - Modified weighted stair climb: 15 rep | *20’ Exercises*  *TABATA 3 series*  *20’’ rest between exercises*  *60’’ rest between exercises* |
